# Green quality by design HPLC approach for the simultaneous determination of Bilastine and Montelukast

**DOI:** 10.1186/s13065-023-00953-y

**Published:** 2023-05-02

**Authors:** Aya Roshdy, Randa Abdel Salam, Ghada Hadad, Fathallah Belal, Heba Elmansi

**Affiliations:** 1 Department of Pharmaceutical Chemistry, Faculty of Pharmacy, Horus University-Egypt, New Damietta, Egypt; 2 Department of Pharmaceutical Chemistry, Faculty of Pharmacy, Suez Canal University, Ismailia, Egypt; 3 Department of Pharmaceutical Analytical Chemistry, Faculty of Pharmacy, Mansoura University, Mansoura, 35516 Egypt

**Keywords:** Quality by design, Bilastine, Montelukast, Stability, HPLC

## Abstract

For the simultaneous estimation of two co-formulated antihistaminic drugs (Bilastine and Montelukast), a novel and eco-friendly reversed-phase HPLC approach with both diode array and fluorescence detection modes was designed. Rather than using the routine methodology, the Quality by Design (QbD) approach was adopted to speed up the method development and to test robustness of the method. To evaluate the effect of variable factors on chromatographic response, a full factorial design was used. The chromatographic separation was performed using isocratic elution on the C18 column. The mobile phase consists of 92% methanol, 6% acetonitrile, and 2% phosphate buffer with 0.1 (v/v) triethylamine adjusted to pH 3, it was pumped at a flow rate of 0.8 mL/min with an injection volume of 20 μL. The developed stability indicating HPLC approach was used to assess the stability of montelukast (MNT). It was subjected to a variety of stress conditions, including hydrolytic (acid–base), oxidative, thermal, and photolytic stress conditions. All of these conditions were found to have relevant degradation pathways. Under the described experimental conditions, MNT degradation followed pseudo-first-order kinetics. The kinetic parameters of its degradation (rate constant and t_1/2_) were calculated and a proposal for the degradation pathway was postulated.

## Introduction

Seasonal allergic rhinitis, commonly referred to as hay fever, affects millions of people worldwide. Symptoms include sneezing, nasal congestion, runny nose, and itching in your nose, the roof of your mouth, throat, eyes, or ears. The most common seasonal triggers are pollen grains and mold spores in the air, which trigger a chain reaction in your immune system [1]. The combination of bilastine and montelukast has been recently used to relieve the symptoms of seasonal allergic rhinitis [2].

**Fig. 1:**
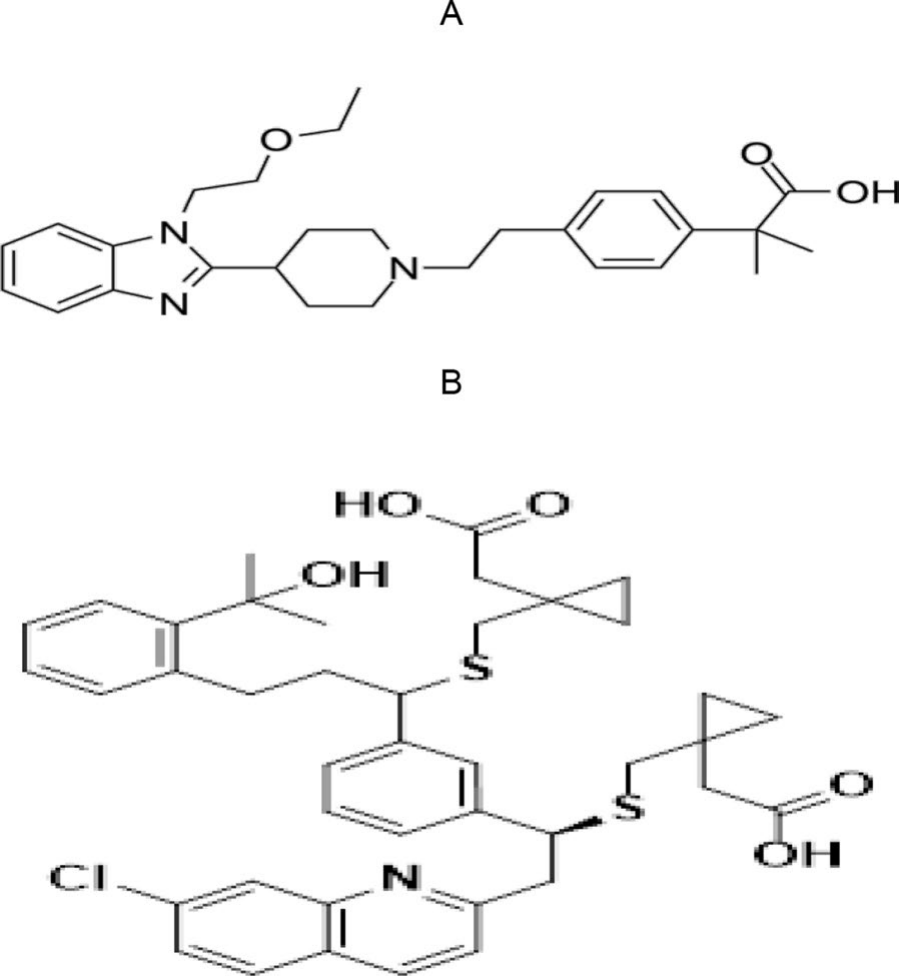
The structural formulae of the studied drugs. **A** Bilastine. **B** Montelukast

Bilastine (BIL) is, 2-[4-(2-(4-(1-(ethoxy ethyl)-1H-benzimidazole-2-yl) piperidine-1-yl) ethyl) phenyl]-2-methyl propionic acid (Fig. 1-1), is a unique second-generation H1 antihistamine that has been found to cause no sedation or cardiotoxicity in clinical studies or post-marketing experience. It was established to treat the symptoms of allergic rhino conjunctivitis and urticaria [3]. variable approaches for determining BIL have been investigated, including spectrophotometric [4], spectrofluorometric [5], and chromatographic methods [6–8].

Montelukast (MNT) is 2-[1-({[(1R)-1-{3-[(E)-2-(7-chloroquine in-2-yl)-ethenyl]phenyl}-3-[2-(2-hydroxypropyl-2 yl)phenyl]propyl]sulfanyl}methyl)cyclopropyl] acetic acid pyl] acetic acid [9] (Fig. 1B).

It is a powerful leukotriene receptor antagonist and is used to treat asthma and seasonal allergic rhinitis [10].

Variable approaches for determining MNT have been investigated, including spectrophotometric [11], spectrofluorometric [12] and chromatographic methods [13–15].

To give additive benefits, it is recommended that these two drugs be used together in reducing SAR symptoms and allergic rhinitis-associated asthma.

The analytical approaches for determining both drugs have been recently reported. These methods include spectrophotometry [16] and the HPLC method [17–23]. However, the proposed method has the advantages of being more sensitive than the reported methods for both drugs, greener as minimum amount of acetonitrile was used compared with these methods and the degradation pathway of montelukast was provided. Additionally, two detectors were investigated during the determination which are fluorescence detection and diode array detection.

There is always a need for essential stability-indicating methods (SIMs) in a modern analytical laboratory. The standards demand that forced degradation tests be carried out under a variety of conditions, including pH, light, oxidation, dry heat, and λ isolation from degradation products. Furthermore, stability testing methodologies and kinetic studies on drug disintegration are required for quality control and to estimate the expiration date of pharmaceuticals [24].

Analytical Eco-Scale and GAPI approaches were used to estimate the suggested analytical method's greenness.

In this study, a HPLC approach for the simultaneous analysis and quantification of bilastine and montelukast is developed which has the advantages of being more sensitive, and greener compared with reported methods and has a wider range.

With a small number of trials, the design of the experiment (DOE) was used to explore the effect of a variety of conditions on specific responses, as well as their interactions. As a result, the volume of organic solvents and chemicals used during the research was reduced. In addition to applying the approach to dosage forms, we aimed to investigate the stability of montelukast under stress conditions.

## Experimental measures

### Apparatus, materials, solvents, and reagents

- Shimadzu Prominence Series LC-2030 C (Shimadzu., Kyoto, Japan) with a Quaternary RS pump, RS auto-sampler, Thermostated RS column compartment, and RS diode array detector (DAD) and fluorescence detector RD-20A.
- Knauer^®^ ODS (150 × 4.6 mm, 5 µm) C18 column.
- All the chromatographic data obtained were manipulated using Lab solutions software.
  Minitab^®^ statistical software was used to perform the factorial design and statistical analysis (release 16 for windows, state college, Pennsylvania, USA).
  Consort NV P-901 pH Meter (Belgium) was used to adjust pH.
- Bilastine (BIL) was kindly supplied by Mash Premiere (El- Obour City, Egypt), and its purity was 99.3%.
- Montelukast (MNT) was kindly donated by Novartis Pharma AG, Egypt, and its purity was 99.2%.
- Organic solvents (HPLC grade) and triethylamine (≥ 99.5%) were bought from the distribution center of Sigma-Aldrich in Cairo, Egypt.
- Orthophosphoric acid (85 percent, w/v) was bought from Riedel de Häen (Seelze, Germany).
- Sodium dihydrogen phosphate and sodium hydroxide were obtained from ADWIC Co. (Cairo, Egypt).
- Each prepared tablet contained (20 mg of BIL and 10 mg of MNT), 20 mg of talc powder, 15 mg of maize starch, 15 mg of lactose, and 10 mg of magnesium stearate.

### Standard solutions and mobile phase

#### Standard solutions

To obtain 100.0 µg/mL standard solutions of each drug, BIL and MNT samples were accurately weighed (0.010 g) and dissolved in methanol in 100.0 mL volumetric flasks. The stock solutions were kept in the refrigerator and were found to be stable for two weeks, MNT should be protected from light.

#### Mobile phase

The mobile phase consists of 92% methanol, 6% acetonitrile, and 2% phosphate buffer with 0.1% v/v triethylamine adjusted to pH 3. Before being filtered via 0.45 µm membrane filters, the fluid was ultrasonicated.

### Procedures

#### Constructing the calibration graphs

Serial dilutions of each of the BIL and MNT standard solutions were prepared with final concentrations of 1.0–50.0 µg/mL for BIL and 3.0–40.0 µg/mL for MNT with a PDA-detector. For MNT solution with the FLU-detector was (0.2–5.0 µg/mL). Serial concentrations were transferred into a series of 10 mL volumetric flasks, and completed to the mark with the mobile phase and mixed well. The mobile phase flowed at a rate of 0.8 mL/min and 20 µL aliquots were injected in triplicate under the optimum chromatographic conditions. The calibration curves for both analytes were constructed by plotting the average peak area against the final drug concentration (c) in µg/mL and the corresponding regression equation for each drug was derived.

#### Analysis of pharmaceutical preparations

Ten tablets were prepared by mixing powder proportions corresponding to the weight of one tablet (0.02 g of BIL and 0.01 g of MNT). A quantity of the powder equivalent to one tablet was transferred into a flask then adding approximately 50.0 mL of methanol was. The flask was sonicated for 30 min before being completed with methanol in 100 mL volumetric flasks and then filtered with a syringe filter. Further dilution with distilled water was carried out to produce a working standard solution, which was then assessed using the general procedure described before under 2.3.1. The nominal content was calculated using either previously generated calibration graphs or using the corresponding regression equation.

#### kinetic studies of degradation of MNT

According to ICH guidelines [25], different stress conditions were applied to aliquots of MNT solution (10 µg/mL). For the acidic degradation study, the appropriate volumes of MNT were transferred into a series of 10 mL volumetric flasks and exposed to the stated acid (1 mL 0.3 M HCl) at different time intervals (0–3 h) at 40 °C.

Similarly, for basic degradation, it was exposed to alkali (0.3 M NaOH) at different time intervals (0–3 h) at 40 °C.

For thermal degradation, the flasks were kept in a water bath heated at different temperatures (25, 35,45,60, and 70 °C) for 1 h.

For photolytic degradation, UV lamb was used at time intervals (0.0–4 h) in a wooden cabinet at a distance of 10 cm.

For oxidative degradation, 10% v/v H_2_O_2_ was added to the flasks at time intervals (0.0–3 h) at room temperature.

Samples were transferred, neutralized, or evaporated under nitrogen (if necessary), diluted with deionized water, and evaluated sequentially using the developed HPLC method, with the remaining MNT concentrations estimated using the corresponding regression equation.

## Results and discussion

### Optimizing the method

#### The wavelength of the diode array detector (PDA detector)

The diode array scanning range was from 190 to 400 nm and the detection wavelength was set at 250 nm above the cutoff point of methanol.

#### Excitation/emission wavelengths of fluorescence detector (FLU-detector)

A spectrofluorometer was used to individually scan 10 µg/mL of each of the BIL and MNT solutions. Only MNT has an emission band at λem = 390 nm (λex = 340 nm) therefore the fluorescence detection was carried out at 340/390 nm.

- **The mobile phase composition, pH, and flow rate**
  Each methanol and acetonitrile were tried as organic modifiers, and the optimal combination was found to be a mixture of methanol and acetonitrile, which caused MNT to separate from the column. According to previous trials, decreasing the methanol ratio caused a significant increase in the retention time of MNT with poor sensitivity, hence we used 85 to 92 % (v/v) methanol.
- Factorial design approach
  Chromatographic conditions screening and optimization "Univariate optimization" is a complex procedure that necessitates a large number of experiments to attain the best results. The study only examines one variable at a time while the others were kept constant, which is time-consuming.

Full factorial design is a form of DOE "multivariate optimization" that permits researchers to explore the effect of all parameters while simultaneously varying them, and evaluating the actions of independent elements and their interactions [26]. The method of factorial design requires few runs for each investigated parameter, allows identifying influential process parameters without time consuming and costly tests.

QbD allows for a more efficient and effective development process, reducing the time required for method development and optimization. This is achieved by identifying the critical parameters and their impact on the responses, which allows for a more focused and targeted approach to method development. it also ensures that the method is robust, and any variations in the parameters will not affect the quality of the final results. This is achieved by identifying and controlling the critical parameters and understanding the effect of changes in these parameters on the responses.

In this investigation, a full factorial design with two levels and four independent factors was used to test and optimize the chromatographic conditions. The percent of methanol, acetonitrile, buffer pH, and flow rate were the most essential elements influencing the HPLC process, and DOE optimized them.

Methanol ratios of 85 and 92% were tested, while acetonitrile ratios varied from 3 and 6%. The pH of potassium dihydrogen phosphate was between 3 and 6. For the analysis of acidic and basic drugs in a reversed-phase system at these pHs, flow rates of 0.8 and 1.2 mL/min were selected. As a result, the two levels were (− 1) for the lower level and (+ 1) for the higher level, with the four independent parameters being percent of methanol (A), percent of acetonitrile (B), buffer pH (C), and flow rate (D) [27].

The interaction of each level on the responses including tailing of montelukast peak (T2) (R1), number of theoretical plates of MNT (NTP) (R2), Run time and resolution between the two drugs (R3) (R4) was studied using a 2^3^ full factorial design that suggested 16 runs. Tables 1 and 2 shows the two levels, independent variables, and dependent variables.

**Table 1:** 2^3^ Experimental factorial designs and their dependent responses for RP-HPLC–UV separation of BIL and MNT mixture

Design order		Experimental factorial design			Dependent responses				
Std order	Run order	%MeOH (A)	%ACN (B)	pH (C)	Flow rate (D)	T2 of MNT	NTP 2 of MNT	Run time	Rs
5	1	85	3	6	0.8	1.42	429	6.1	6.1
6	2	92	3	6	0.8	1.3	1642	3.5	3.5
14	3	92	3	6	1.2	1.3	893	3.6	3.6
15	4	85	6	6	1.2	1.7	661	5	5
4	5	92	6	3	0.8	1.2	3226	7.3	7.3
9	6	85	3	3	1.2	1.65	165	2.9	2.9
1	8	85	3	3	0.8	1.9	151	2.81	2.81
2	9	92	3	3	0.8	1.25	3341	10	10
8	10	92	6	6	0.8	1.12	1261	2.8	2.8
16	11	92	6	6	1.2	1.28	1135	2.56	2.56
3	12	85	6	3	0.8	1.56	328	3.85	3.85
13	14	85	3	6	1.2	1.41	1417	6.48	6.48
11	15	85	6	3	1.2	1.36	591	5.20	5.20
12	16	92	6	3	1.2	1.20	2159	5.90	5.90
10	17	92	3	3	1.2	1.20	2367	8.40	8.40
7	18	85	6	6	0.8	1.40	918	5.90	5.90

MeOH%: MeOH% (v/v) in the organic mobile phase (low level 85% and high level 92%)

%ACN: ACN% (v/v) in the organic mobile phase (low level 3% and high level 6%)

pH: Aqueous mobile phase pH (low level 3 and high level 6)

Flow rate (low level 0.8 and high level 1.2)

Rs1: resolution between BIL & MNT

**Table 2:** Parameters of system suitability of the proposed HPLC method for the determination of BIL and MNT

Parameter	UV chromatographic value		Fluorescence chromatographic value
	BIL	MNT	MNT
No theoretical plates, N	1443	1093	1103
Capacity factor, km	0.26	0.95	0.9
Selectivity factor, α	3.65		
Resolution, R_s_	3.2		
Retention time (t_R_), min	2.4	3.7	3.6
Tailing factor (T)	1.45	1.27	1.27

Where: Number of theoretical plates (N) = $5.54\left( {\tfrac{{{\text{tR}}}}{{\text{Wh/2}}}} \right)^{2}$

Resolution (R_s_) = $$\tfrac{{2\Delta {\text{tR}}}}{{{\text{W}}1\; + \;{\text{W}}2}}$$

Capacity factor `) = $$\frac{{\mathrm{t}}_{\mathrm{R}}-{\mathrm{t}}_{\mathrm{m}}}{{\mathrm{t}}_{\mathrm{m}}}$$

The electivity (α) = $$\frac{{\mathrm{k}`}_{2}}{{\mathrm{k}`}_{1}}$$

Tailing factor (T) = $$\frac{{W}_{0.05}}{2d}$$

The significance of independent factors was determined using an approximated Fisher Statistical Test for Variance Analysis (ANOVA) model [28] that was applied to the responses to investigate the effect of these independent factors on the responses as well as their interactions. The equation for the four-factor experimental design is as follows:

$$\begin{gathered} {\text{R }} = \, \beta 0 \, + \, \beta 1{\text{A }} + \, \beta 2{\text{B }} + \, \beta 3{\text{C }} + \, \beta 4{\text{D}} + \, \beta 2{\text{AB }} + \, \hfill \\ \;\;\beta 2{\text{AC }} + \, \beta 2{\text{BC}} + \, \beta 2{\text{BD }} + \, \beta 2{\text{A}}2 \, + \, \beta 2{\text{B}}2 \, + \, \beta 2{\text{C}}2 + \, \beta 2{\text{D}}2, \hfill \\ \end{gathered}$$

where R stands for a response, β stands for regression coefficients, and A, B, C, and D stand for methanol %, acetonitrile %, buffer pH, and flow rate, respectively.

To be sure that the best possible circumstances are achieved, the Minitab response optimizer determines the composite desirability of a response (D), which varies from zero to one, and determines whether the responses are within reasonable limits. Zero is not acceptable because it indicates that many of the responses are outside their acceptable ranges, whereas one indicates that the conditions obtained are ideal, hence its value should be one or close to one.

**Fig. 2:**
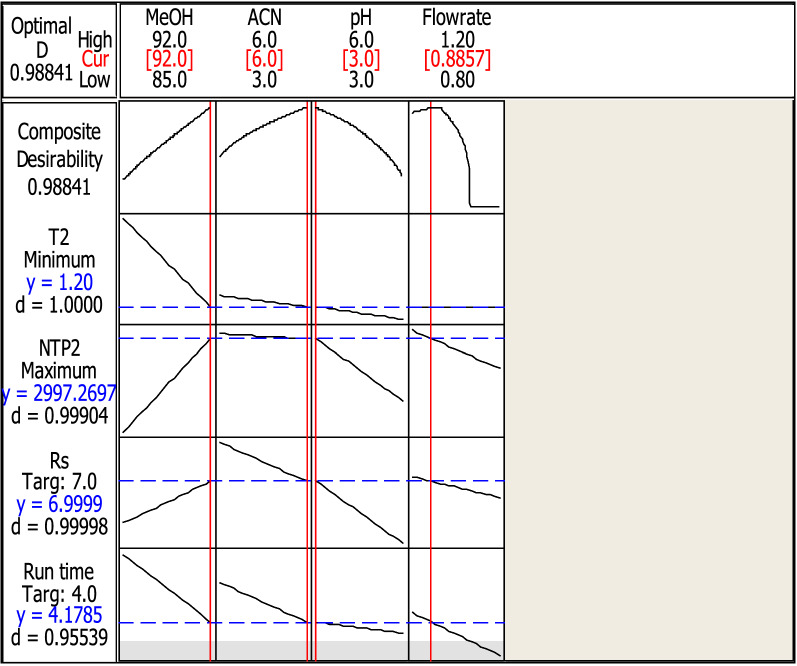
2^3^ Full factorial design optimization plot for the proposed method

The full factorial design yielded the best results; thus, the optimization plot (Fig. 2) demonstrates how the four variables and their interactions affect the composite desirability and response to achieve the best results.

**Fig. 3:**
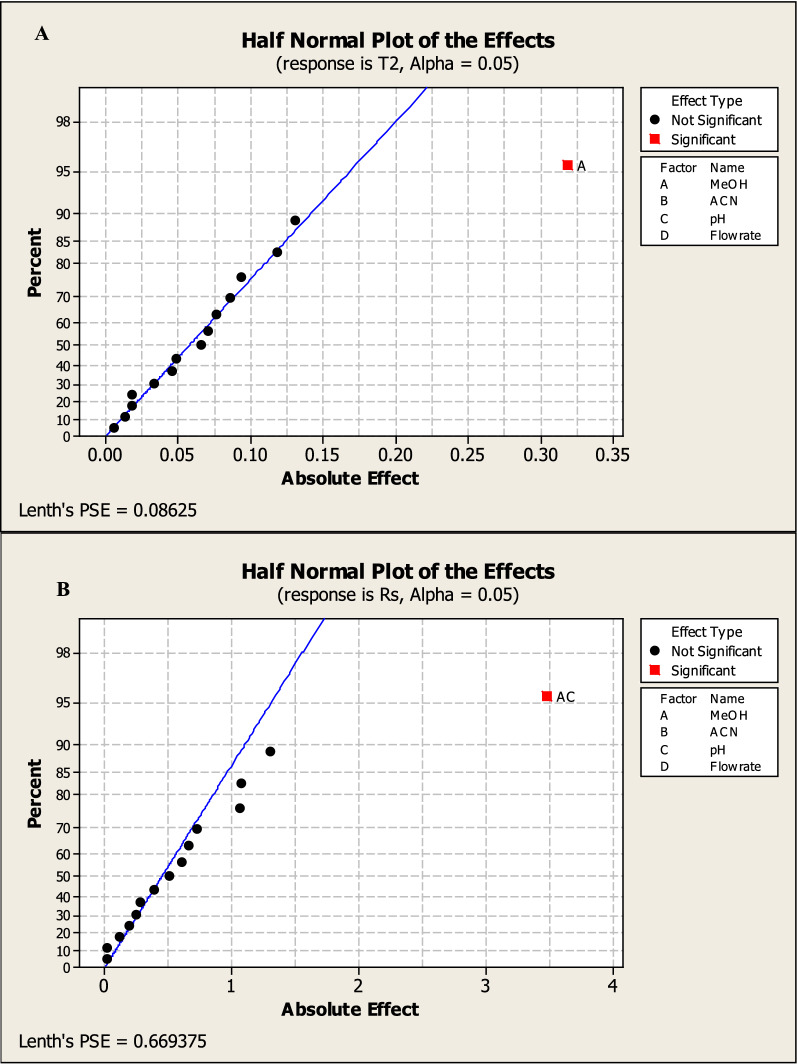

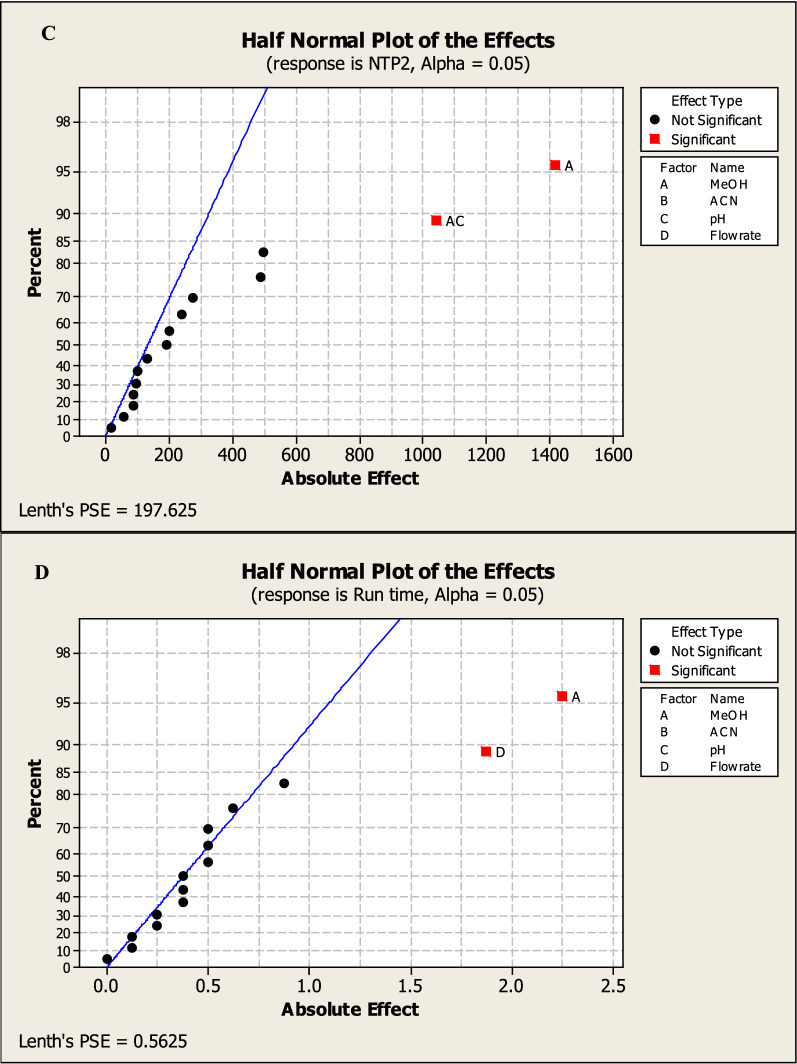
2^3^FFD half normal plots for chromatographic responses by data means type where **A** response to T2, **B** response to Rs, **C** response to NTP 2 and **D** response to Run time

**Fig. 4:**
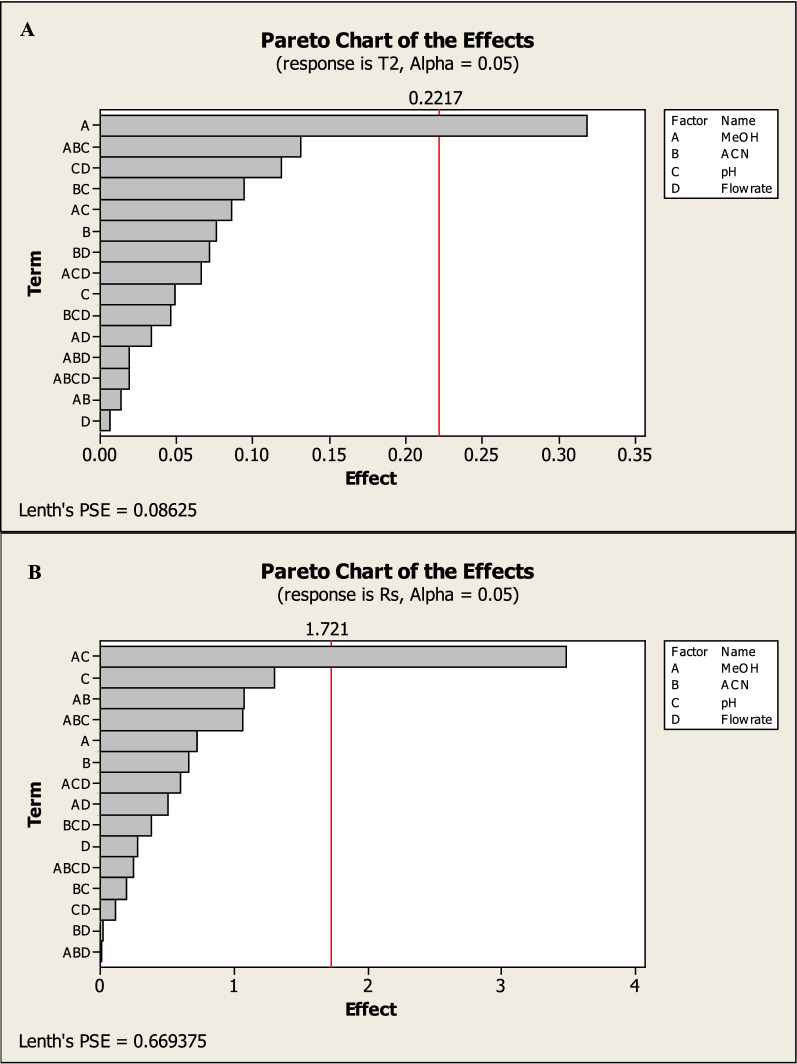

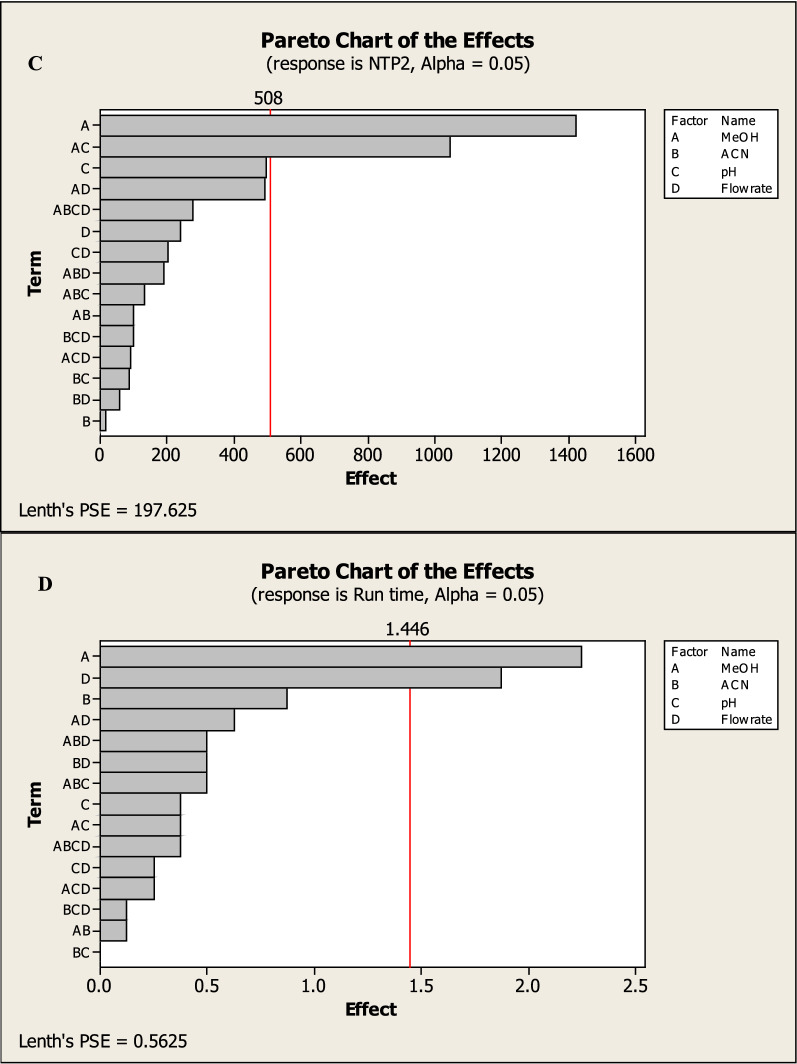
2^3^FFD Pareto charts plots of the effects on the chromatographic responses at alpha = 0.05, where **A** response to T2, **B** response to Rs, **C** response to NTP 2 and **D** response to Run time

Factorial design can also provide half-normal plots and Pareto charts (Figs. 3, 4) show that methanol % (A) has a significant effect on the tailing of MNT (R1), methanol% (A) and methanol with pH (AC) have a significant effect on NTP of MNT (R2), also methanol with pH (AC) has a significant effect on resolution between the two drugs (R3) while methanol% (A) and flow rate (D) have a significant effect on run time (R3) with statistically significant effect for a 95% confidence level.

**Fig. 5:**
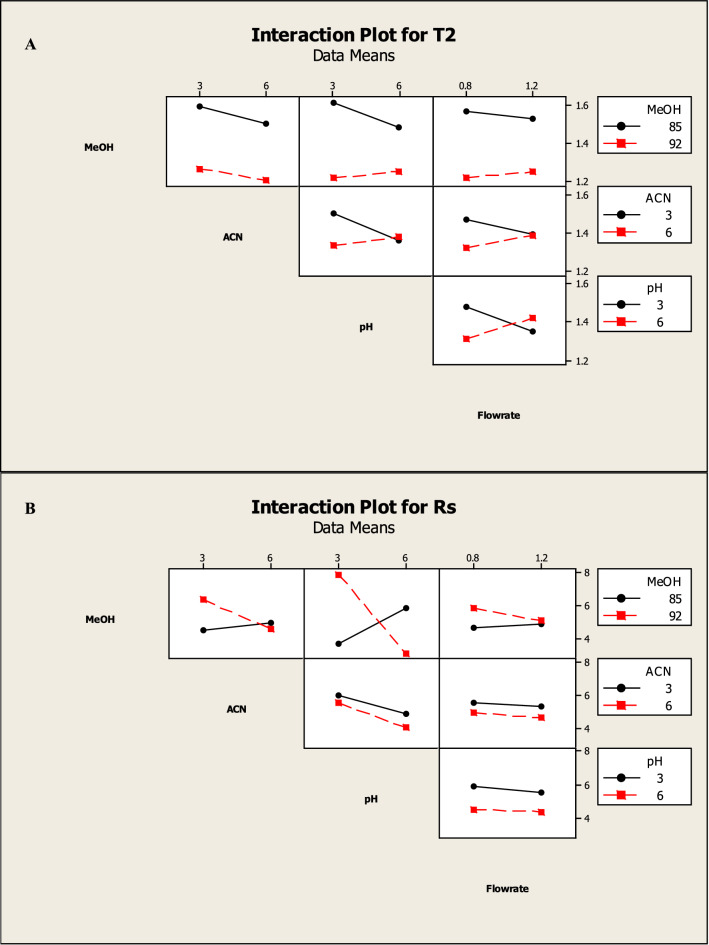

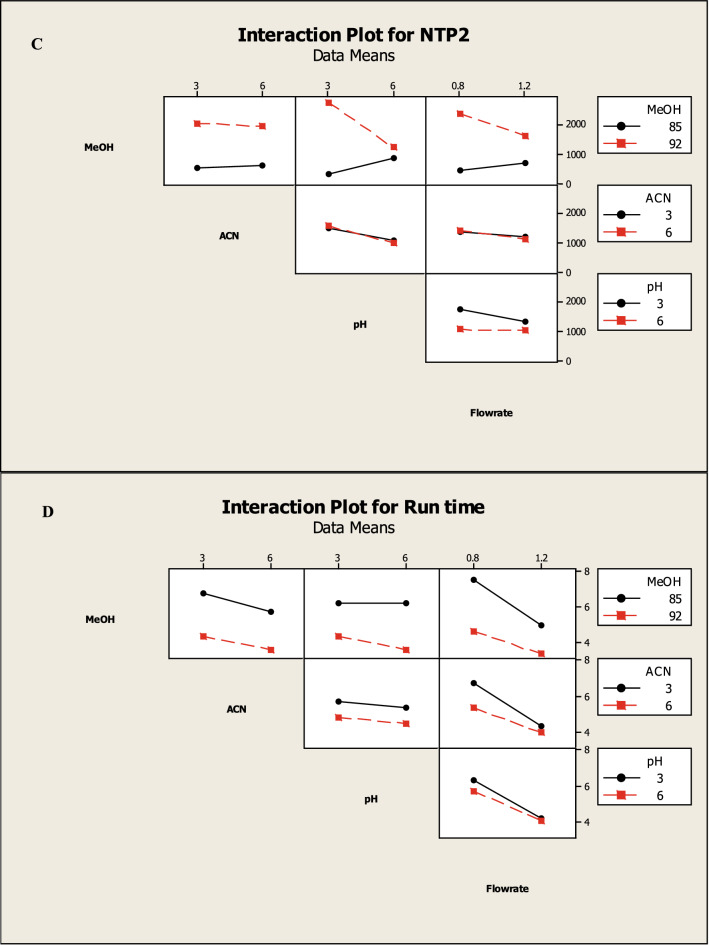
2^3^ FFD interaction plots for chromatographic responses by data means type, where **A** response to T2, **B** response to Rs. **C** response to NTP 2 and **D** response to Run time

**Fig. 6:**
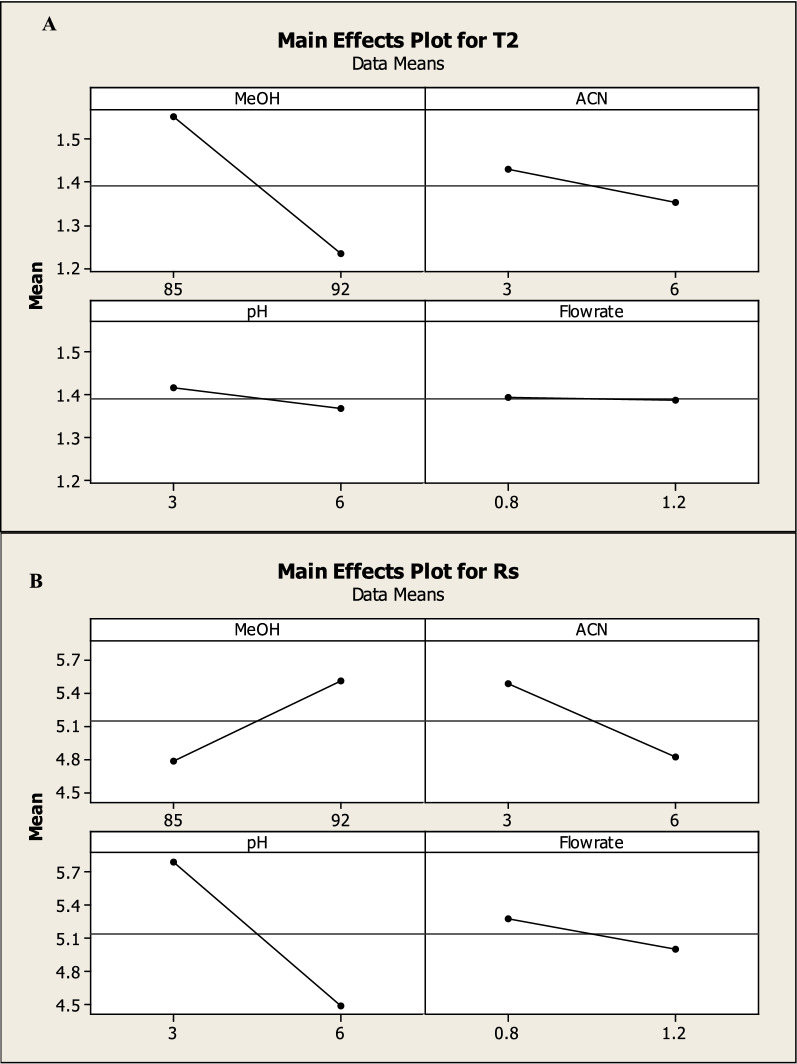

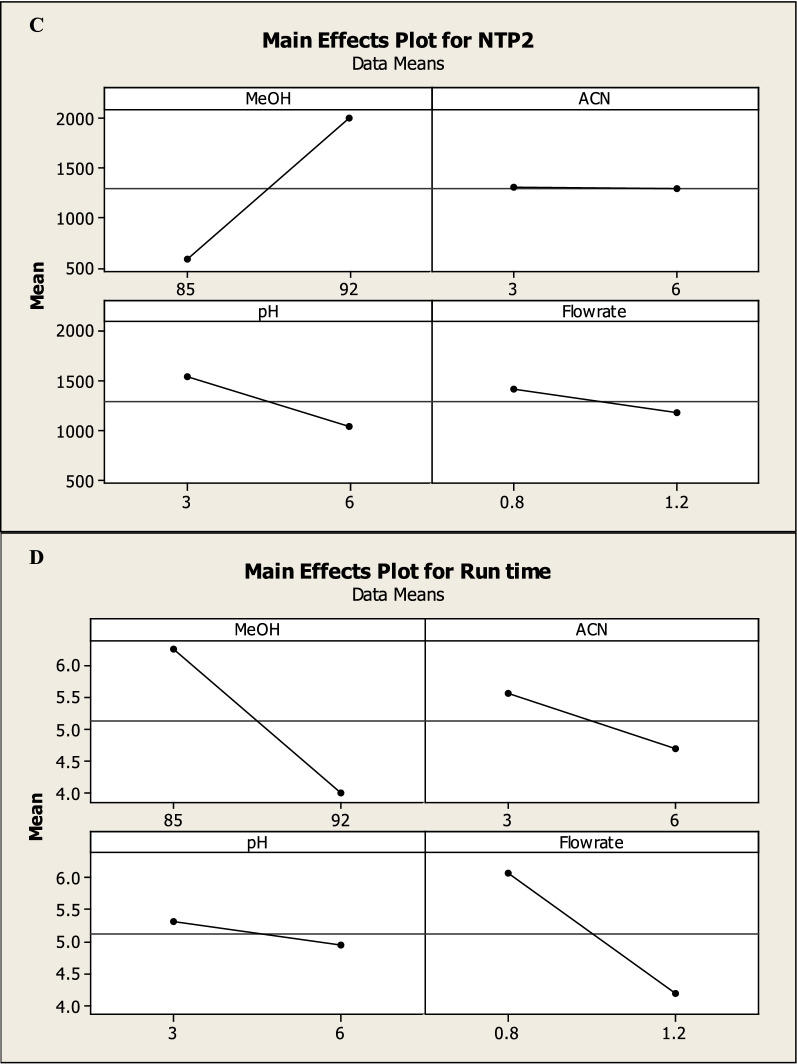
2^3^ FFD main effect plots for chromatographic responses by data means type, where **A**: response to T2, **B**: response to Rs. **C**: response to NTP 2 and **D**: response to Run time

Which were confirmed by the interaction and main effect plots (Figs. 5, 6) show that minimizing the tailing of MNT and the run time, while maximizing the NTP of MNT could be achieved by increasing the percentage of methanol in the mobile phase, reducing the flow rate and using a buffer with pH 3.

**Fig. 7:**
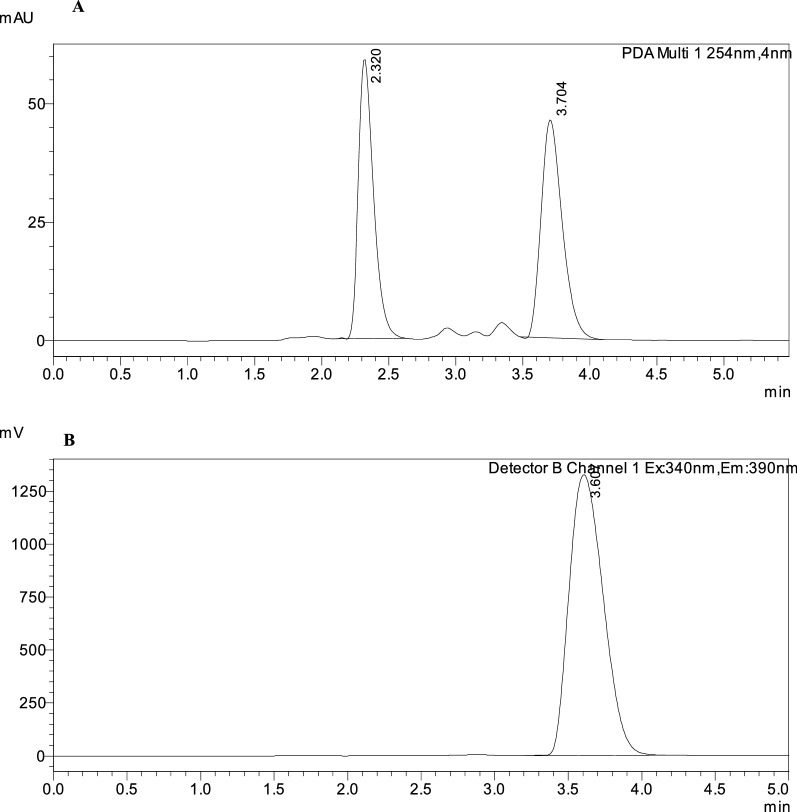
1: Typical chromatogram of the studied drugs under the described chromatographic conditions with a PDA detector at 254 nm. 2: Typical chromatogram of MNT (10 μg/mL) under the described chromatographic conditions with FLU detector at 340/390 nm

Thereafter, the mobile phase was obtained using a mixture of 92 (v/v) % methanol, 6% acetonitrile, and 2% phosphate buffer with 0.1 percent v/v triethylamine adjusted to pH 3 according to DOE (Fig. 7).

### Validation parameters

Validation parameters were evaluated following the International Conference on Harmonization (ICH) Q2R1 guidelines [25]. After analyzing six concentrations for each of BIL and MNT, regression equations were used to investigate the linearity and ranges for the two drugs. As indicated in Table 3, the recommended HPLC procedure was applied to pure samples of BIL and MNT over the ranges of 1.0–50.0 and 3.0–40.0 µg/mL using the PDA-detector, respectively. and MNT (0.2–5.0 µg/mL) using the FLU detector. For the two drugs, the peak area (y) was plotted against the concentration (c), and the correlation coefficients (r) were determined to be 0.9999. Regression equations of the data were calculated and given as:

$${\text{y }} = {\text{ 99}}0{\text{5}} + {\text{ 27}}0{\text{5}}0{\text{C}}\,\left( {{\text{r }} = {\text{ }}0.{\text{9999}}} \right)\,{\text{for BIL}}$$

$${\text{y }} = {\text{ 66375}} + {\text{ 44426C}}~\,\left( {{\text{r }} = {\text{ }}0.{\text{9999}}} \right)\,{\text{for MNT}}\,{\text{for PDA - detector}}$$

$${\text{y}} = {\text{4725}}0{\text{ }} + {\text{ 4324}}0{\text{ C}}~\,\left( {{\text{r }} = {\text{ }}0.{\text{9998}}} \right)~\,{\text{for MNT}}\,{\text{for FLU - detector}}$$

**Table 3:** Analytical performance data for the determination of the BIL and MNT by the proposed HPLC method

Parameter	PDA detector		Fluorescence detector
	BIL	MNT	MNT
Linearity range (µg/mL)	1.0–50.0	3.0–40.0	0.2–5.0
Intercept (a)	10,712	67,237	883,254
Slope (b)	27,008	44,383	2,210,025
Correlation coefficient (r)	0.9999	0.9998	0.9999
S.D. of residuals (Sy/x)	3646	18,316	1.35
S.D. of intercept (Sa)	2310	12,886	14,691
S.D. of slope (Sb)	89	434	5511
Percentage relative standard deviation, % RSD	0.619	1.21	1.348
Percentage relative error, % Error	0.25	0.49	0.604
Limit of detection, LOD (µg/mL)	0.28	0.96	0.02
Limit of quantitation, LOQ (µg/mL)	0.85	2.9	0.1

The detection limits (DL) and the quantitation limits (QL) were calculated referring to the following equations [25], and the obtained data are shown in Table 3:

$${\text{QL}} = { 1}0{\text{ S}}_{{\text{a}}} /{\text{b}} {\text{DL}} = { 3}.{\text{3 S}}_{{\text{a}}} /{\text{b}}{.}$$

When statistically comparing the results obtained using the suggested method with those obtained from the comparison method [17] as shown in Table 4, it was found that no significant difference regarding accuracy and precision [29]. The comparison method was RP-HPLC using a C18 column and ACN with water as the mobile phase (70:30 v/v, flow rate 0.8 mL/min, UV detection at 215 nm).

**Table 4:** Assay results for the determination of the studied drugs in pure form by the proposed and comparison methods

Compound	Proposed method			Comparison methods [17]
	Amount taken (µg/mL)	Amount found (µg/mL)	% Found	% Found
BIL	1.0	1.00	100.29	101.30
	3.0	2.97	99.09	98.70
	15.0	14.89	99.30	100.43
	20.0	20.01	100.06	
	30.0	30.22	100.74	
	50.0	49.89	99.79	
Mean	99.88			100.14
± S.D	0.62			1.32
t-test	0.31 (2.31)*			
F-test	4.53 (5.41)*			
MNT (PDA detector)	3.0	2.96	2.96	99.06
	7.0	6.86	6.89	100.94
	15.0	14.96	14.96	99.69
	30.0	30.09	30.09	
	40.0	40.65	40.62	
	50.0	49.44	49.48	
Mean	99.59			99.90
± S.D	1.21			0.96
t-test	0.38 (2.31)*			
F-test	1.49 (5.41)*			
MNT (Fluorescence detector)	0.2	0.20	101.65	99.06
	0.7	0.68	98.04	100.94
	1.0	1.00	100.89	99.69
	3.0	3.00	100.13	
	5.0	4.99	99.95	
Mean	100.13			99.90
± S.D	1.35			0.96
t-test	0.34 (2.31)*			
F-test	1.98 (9.01)*			

Each result is the average of three separate determinations

^*^The figures between parentheses are the tabulated t and F values at *P* = 0.05 [29]

Three separate concentrations of each drug were assessed on three consecutive days between one and three days to evaluate the intraday and interday precisions. Table 5 summarizes the analytical data.

**Table 5:** Precision data for the determination of the studied drugs by the proposed HPLC method

Drug	Conc (μg/mL)	Intra-day			Inter-day		
		Mean ± S.D	%RSD	% error	Mean ± S.D	%RSD	% error
BIL	10.0	99.32 ± 0.65	0.65	0.38	100.51 ± 0.99	0.98	0.57
	20.0	100 ± 1.62	1.62	0.94	100.53 ± 1.5	1.49	0.86
	30.0	98.77 ± 0.77	0.78	0.45	100.07 ± 0.3	0.3	0.17
MNT (PDA detector)	10.0	99.36 ± 1.15	1.16	0.67	99.71 ± 1.37	1.37	0.79
	20.0	100.3 ± 1.02	1.01	0.59	99.87 ± 1.79	1.79	1.03
	30.0	98.83 ± 0.42	0.42	0.24	100.44 ± 1.19	1.18	0.68
MNT (Fluorescence detector)	1.0	100.3 ± 1.3	1.36	0.78	99.4 ± 0.8	0.83	0.44
	2.0	100.4 ± 0.9	0.92	0.53	99.8 ± 0.5	0.51	0.29
	3.0	99.6 ± 0.6	0.74	0.43	100.25 ± 0.35	0.36	0.21

Each result is the average of three separate determinations

**Fig. 8:**
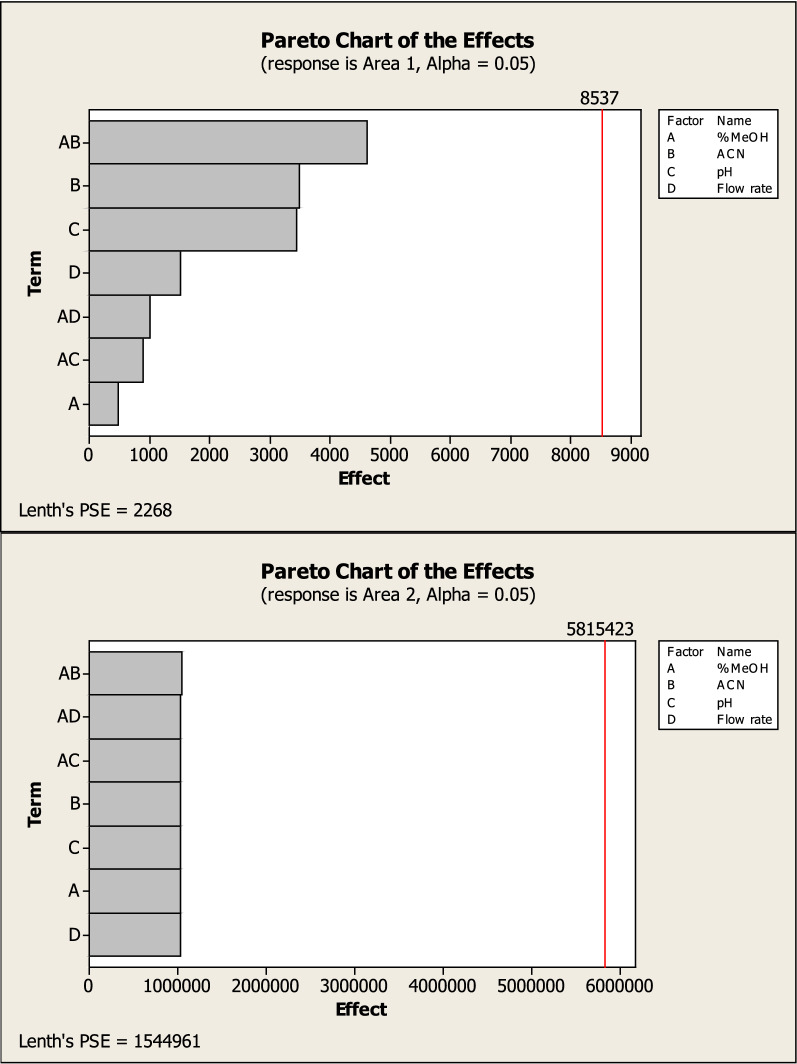
Pareto charts plots for robustness of the effects on the chromatographic responses at alpha = 0.05

A half-factorial design with eight experiments was used to test the robustness of the proposed HPLC method. The robustness of the method was demonstrated by varying the chromatographic conditions such as methanol (92 ± 0.2%), ACN (6 ± 0.1), pH (3 ± 0.2), and flow rate (0.8 ± 0.1 min/mL). As seen in Table 6 and Fig. 8, such minor alterations had little effect on drug resolution, confirming the method's robustness.

**Table 6:** Robustness of the proposed method by half factorial design using 10 µg/mL of each of BIL and MNT

Design order		Experimental factorial design				Dependent responses	
Std order	Run order	%MeOH	CAN	pH	Flow rate	Area 1	Area 2
3	1	91	6.5	2.8	1.2	5,25,951	4,577,528
8	2	93	6.5	3.2	1.2	5,23,287	4,48,416
2	3	93	5.5	2.8	1.2	5,28,840	4,57,504
7	4	91	6.5	3.2	0.8	5,30,803	4,62,550
4	5	93	6.5	2.8	0.8	5,23,246	4,56,989
1	6	91	5.5	2.8	0.8	5,25,330	4,52,036
6	7	93	5.5	3.2	0.8	5,33,911	4,68,304
5	8	91	5.5	3.2	1.2	5,29,164	4,47,743

### Applications

#### Application to prepared tablets and synthetic mixtures

The results obtained using the proposed approach for assessing BIL and MNT in prepared tablets and synthetic mixtures were statistically compared to the results of a previously reported method. [17]. Tables 7, 8 demonstrates that statistical analysis using Student's t-Test and Variance Ratio F-test [29], revealed no significant differences between the two methods regarding accuracy and precision.

**Table 7:** Assay results for the determination of the studied drugs in laboratory-prepared mixtures of their pharmaceutical ratios by the proposed HPLC method

Synthetic mixture	Amt. taken (μg/mL)		% Found	
	BIL	MNT	BIL	MNT
1	10.0	5.0	99.78	98.61
2	20.0	10.0	99.34	99.21
3	30.0	15.0	100.80	101.72
4	50.0	25.0	99.83	99.56
Mean %			99.94	99.78
± S.D			0.62	1.36

Each result is the average of three separate determinations

^*^The figures between parentheses are the tabulated t and F values at P = 0.05 [29]

**Table 8:** Assay results for the determination of the studied drugs in their prepared tablets by the proposed HPLC method

	Proposed method						Comparison method	
	Amount taken (µg/mL)		Amount found (µg/mL)		% Found		% Found	
	BIL	MNT	BIL	MNT	BIL	MNT	BIL	MNT
	10.0	5.0	10.15	4.95	101.53	99.07	100.79	100.85
	20.0	10.0	19.77	10.07	98.85	100.70	98.95	98.87
	40.0	20.0	40.07	19.98	100.19	99.88	100.39	100.42
Mean					100.19	99.88	100.04	100.05
± S.D					1.34	0.82	0.97	1.04
t-test					0.17	0.21	(2.45)*	
F-test					1.91	1.61	(9.28)*	

Each result is the average of three separate determinations

^*^The figures between parentheses are the tabulated t and F values at P = 0.05 [29]

#### Degradation kinetic studies

**Fig. 9:**
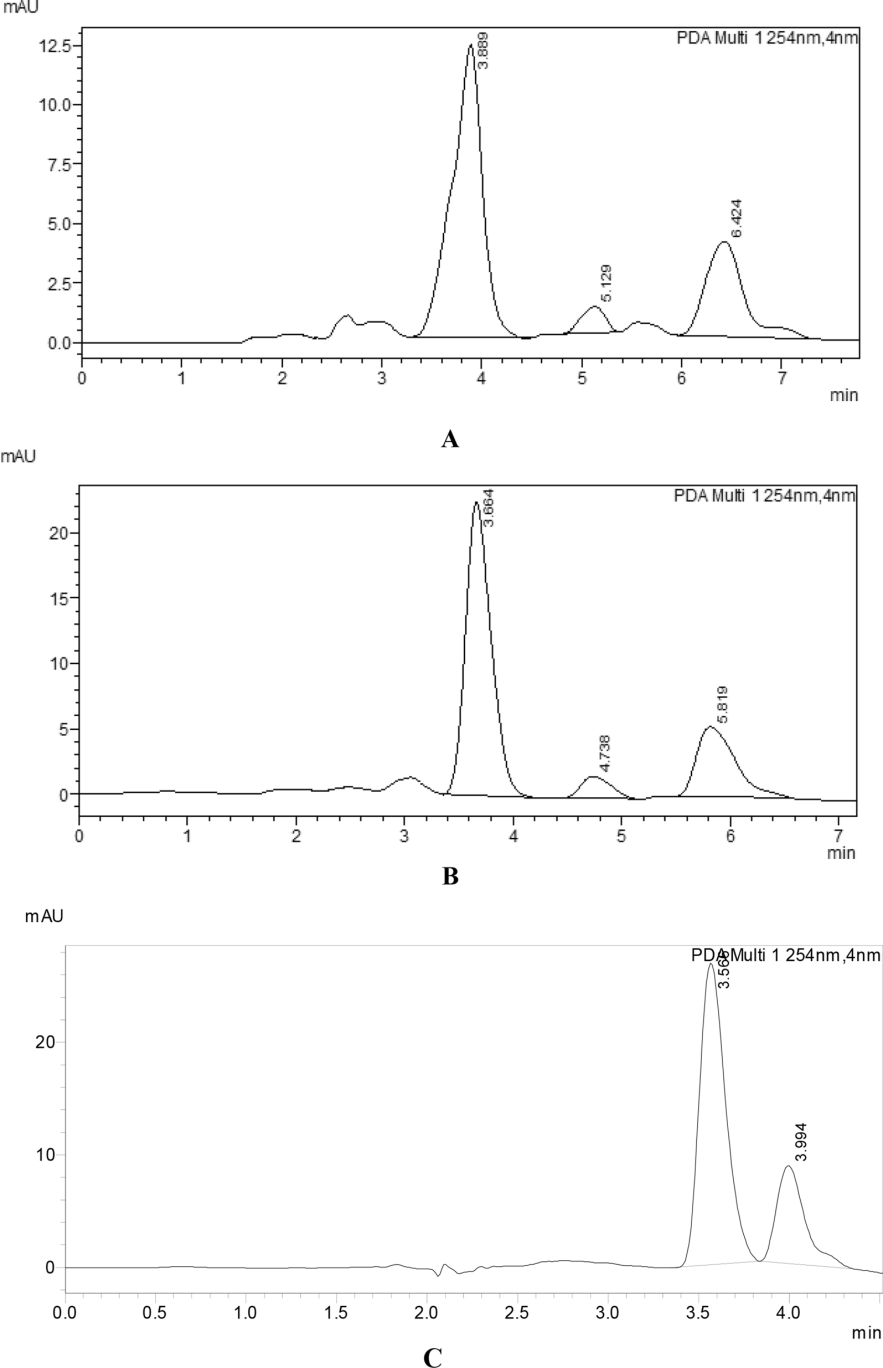

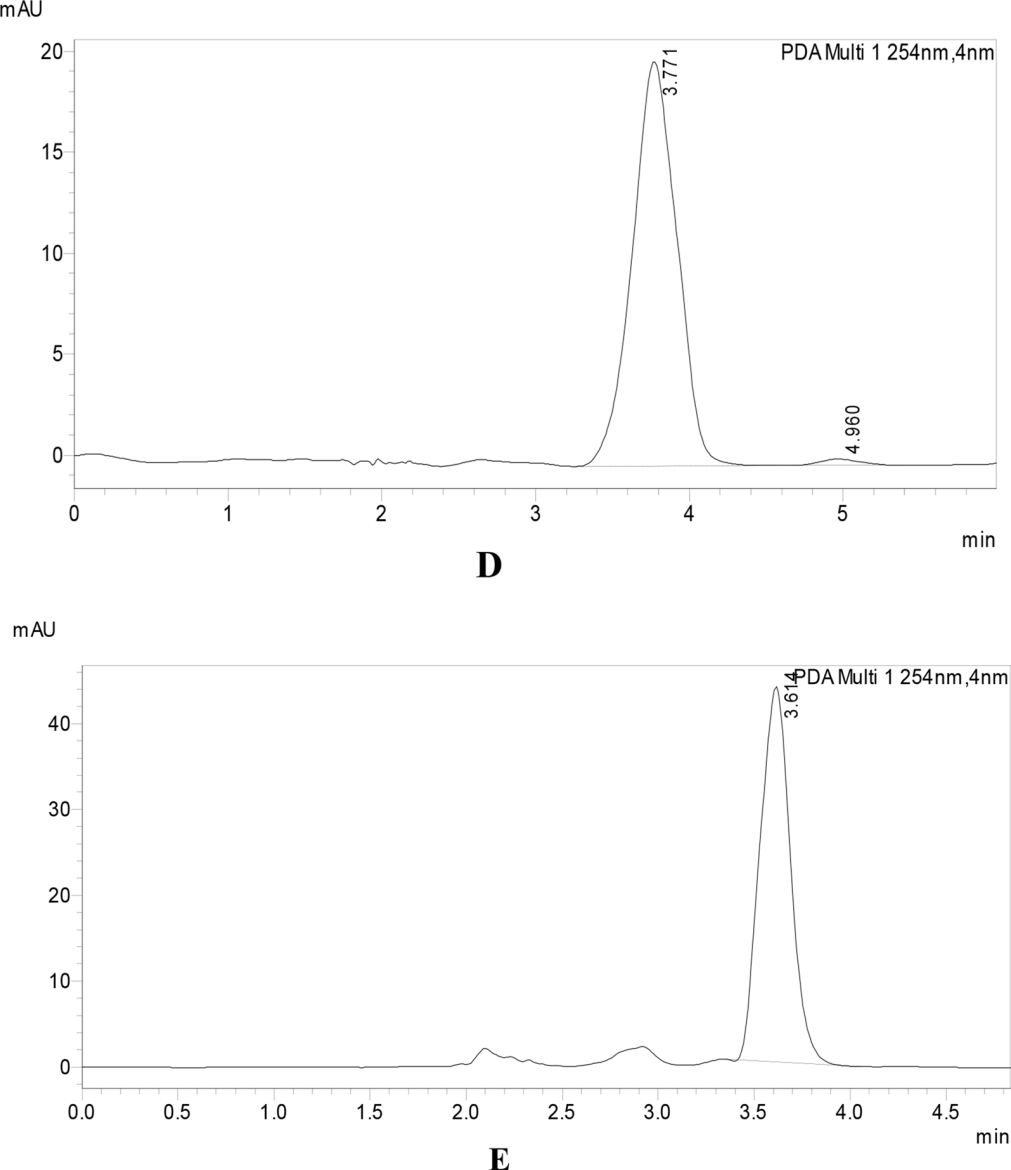
HPLC chromatogram of 10 µg/mL of MNT after acid-induced degradation (**A**), alkaline-induced degradation (**B**), thermal induced degradation (**C**) oxidative-induced degradation (**D**) and photolytic induced degradation with UV radiation at 254 nm (**E**)

**Fig. 10:**
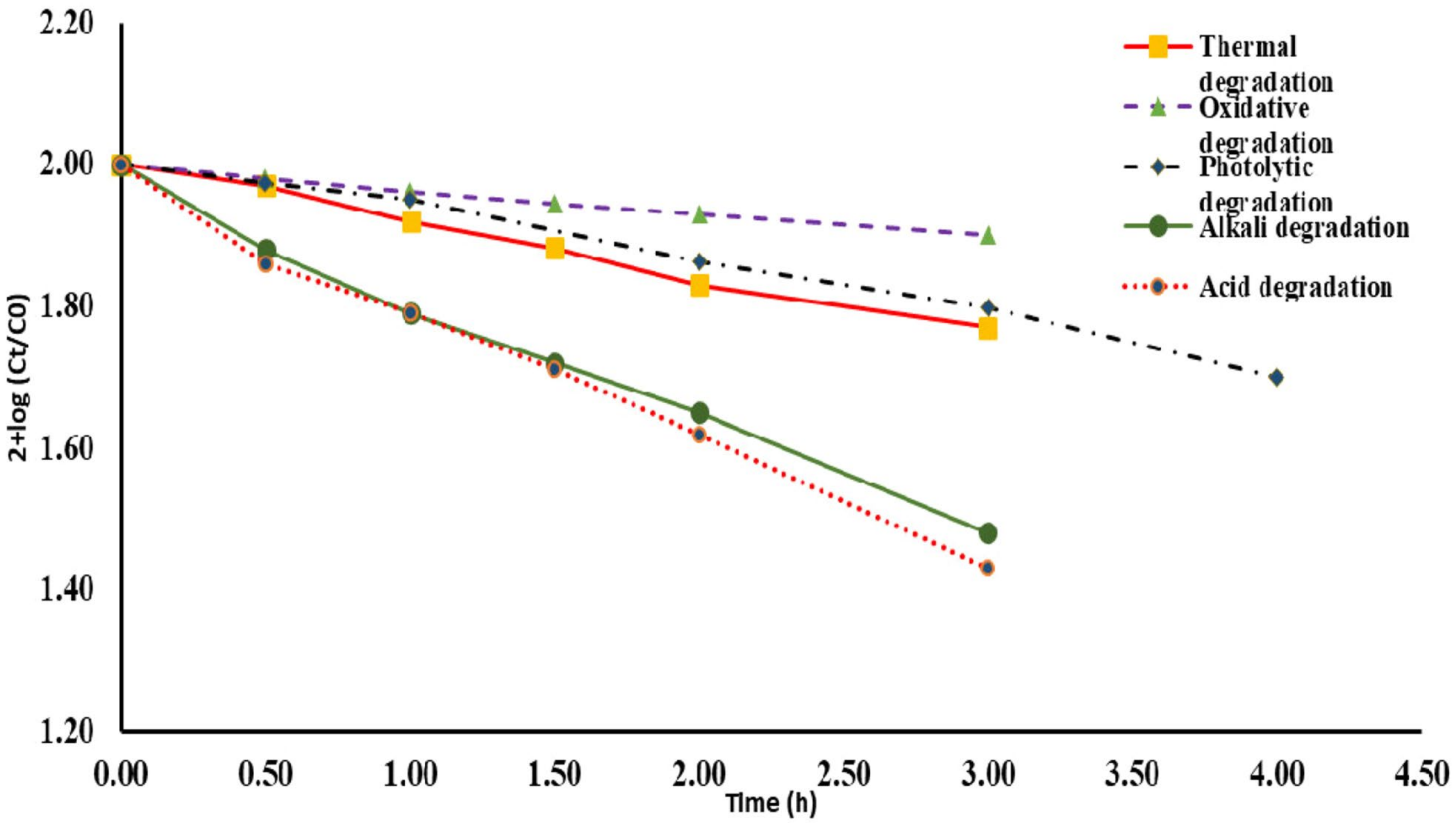
Pseudo first-order kinetic plots for 10 µg/mL of MNT under acid, alkali, thermal, photolytic and oxidative stress conditions

The kinetics of MNT degradation were studied using the proposed approach. Calculating the remaining MNT concentration (Ct) at predefined time intervals was used to get kinetic data. MNT kinetics were investigated in terms of acid, alkali hydrolysis, thermal, photo, and oxidative degradation, where k is the pseudo-first-order rate constant with a negative sign and t is the time. The results obtained match with a reported method for the degradation of MNT under similar stress conditions [15]. The measured reaction rate constants (k) for MNT acid and alkali hydrolysis were 0.414 h^−1^and 0.385 h^−1^, respectively, with half-life times (t1/2) of 1.67 h and 1.8 h. MNT kinetic rate constants for thermal, photolytic, and oxidative degradation pathways were 0.183 h^−1^, 0.17 h^−1^, and 0.075 h^−1^, respectively, with t1/2 values of 3.7, 4.0, and 9.12 h, as shown in Figs. 9, 10.

The acidic and basic degradation pathways are in agreement with those reported in previous studies [15].

In photolytic degradation, it is assumed the drug undergoes dechlorination giving less polar compound [31] (Figs. 9, 10).

In oxidative degradation, the Sulphur atom is supposed to be oxidized to the corresponding sulphoxide giving a more polar compound [32]. This means the drug undergoes degradation by hydrolytic (acid and base), thermal, photolytic, and oxidative actions. The degradant peaks did not interfere with the MNT peak in any of the above conditions, confirming that the approach allowed for the analysis of MNT in the presence of its degradation products (Table 9).

**Table 9:** Kinetic parameter for the Forced degradation of MNT under different stress conditions

Stress condition	Temperature, °C	Time, h	% Degradation	t1/2, h
Acidic (0.3 M HCl)	40	3	37.38	1.67
Basic (0.3 M NaOH)	40	3	31.7	1.8
Thermal	70	3	27.87	3.7
UV radiation (254 nm)	25	3	35.21	4.01
Oxidative (10% H_2_O_2_)	25	3	18.26	9.12

**Fig. 11:**
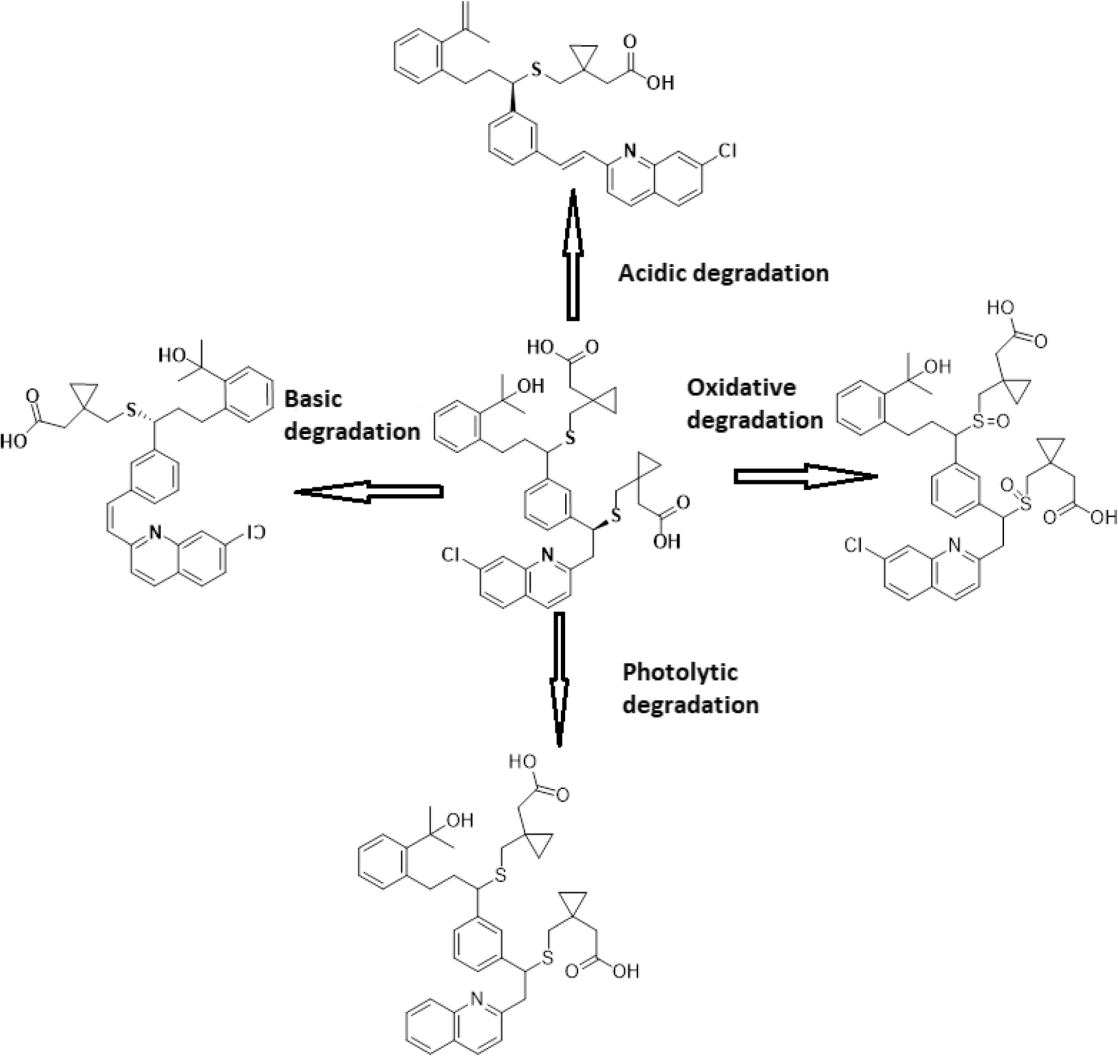
Proposal for the degradation pathways of MNT

A proposal for the degradation pathways is shown in Fig. 11.

### Greenness evaluation methods

**Fig. 12:**
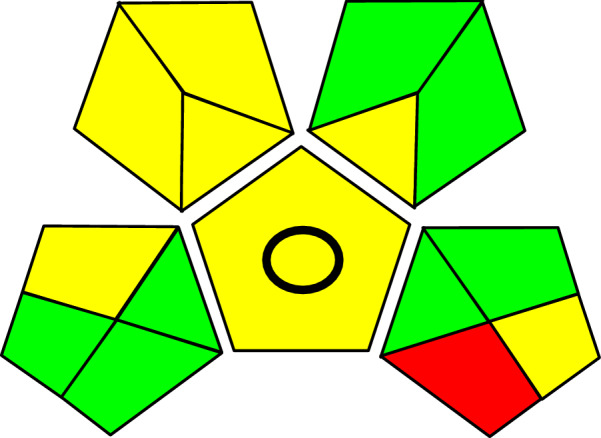
GAPI pictogram for the proposed method

Analytical Eco-Scale and GAPI approaches were used to estimate an analytical method's greenness [33–35]. The proposed approach is a great green method of analysis with few laboratory’s needs, according to the two methods (Table 10; Fig. 12) Using the Analytical Eco-Scale approach, the total number of penalty points for the entire procedure was determined. The results show that the suggested approach worked well for green analysis using the analytical eco-scale of 84.

**Table 10:** Calculation of penalty points for the proposed methods

Reagents	Amount	Signal word	No of pectograms	Sub-total PP
1. MeOH	10–100 mL	Danger = 2	3.00	6.00
2. ACN	< 10 mL = 1	Danger = 2	1.00	2.00
3. Pot. Dihyd. Ph	< 10 mL = 1	0	0.00	0.00
4. Phosphoric.a	< 10 mL = 1	Danger = 2	1.00	2.00
5. TEA	< 10 mL = 1	Danger = 2	1.00	2.00
Total PP for reagents				12.00
Imstrument	HPLC = 2			2.00
Occupational hazard	Not emit vap and gases = 0			0.00
Waste	1–10 mL = 2			2.00
Total PP for instruments				4.00
Total PP				16.00
Analytical Eco-Scale total score:				**84.00**

## Conclusion

A quality-by-design approach to the HPLC method with a Diode array and fluorescence detection modes was designed to determine BIL and MNT in their tablets and to determine MNT in presence of its forced degradation products. MNT is susceptible to acid, alkali hydrolysis, heat, UV, and oxidative-induced degradation. Furthermore, degradation rates were investigated, and it was found that they followed pseudo-first-order kinetics. At three separate levels, a multivariant investigation of various essential process variables was conducted, including % of methanol, % of acetonitrile, and buffer pH. The method is green, precise, accurate, and robust, according to statistical analysis of data. Factorial design provides the most run efficient (economical) data collection plan to learn the relationship between your response variables which allows the method to be used further for routine analysis in quality control labs in the pharmaceutical industry.

## Data Availability

All data analyzed during this study are included in this published article and raw data are available from the corresponding author upon reasonable request.

## Abbreviations

ACN: Acetonitrile
BIL: Bilastine
DAD: Diode array detector
DL: Detection limit
DOE: Design of experiment
GAPI: Green analytical procedure index
NTP: Number of theoretical plate
MNT: Montelukast
QL: Quantitation limit
QbD: Quality by design
% RSD: Percentage relative standard deviation.
SAR: Seasonal allergic rhinitis
SD: Standard deviation.
SIMs: Stability indicating methods
